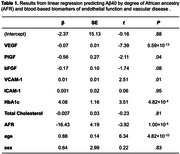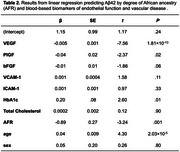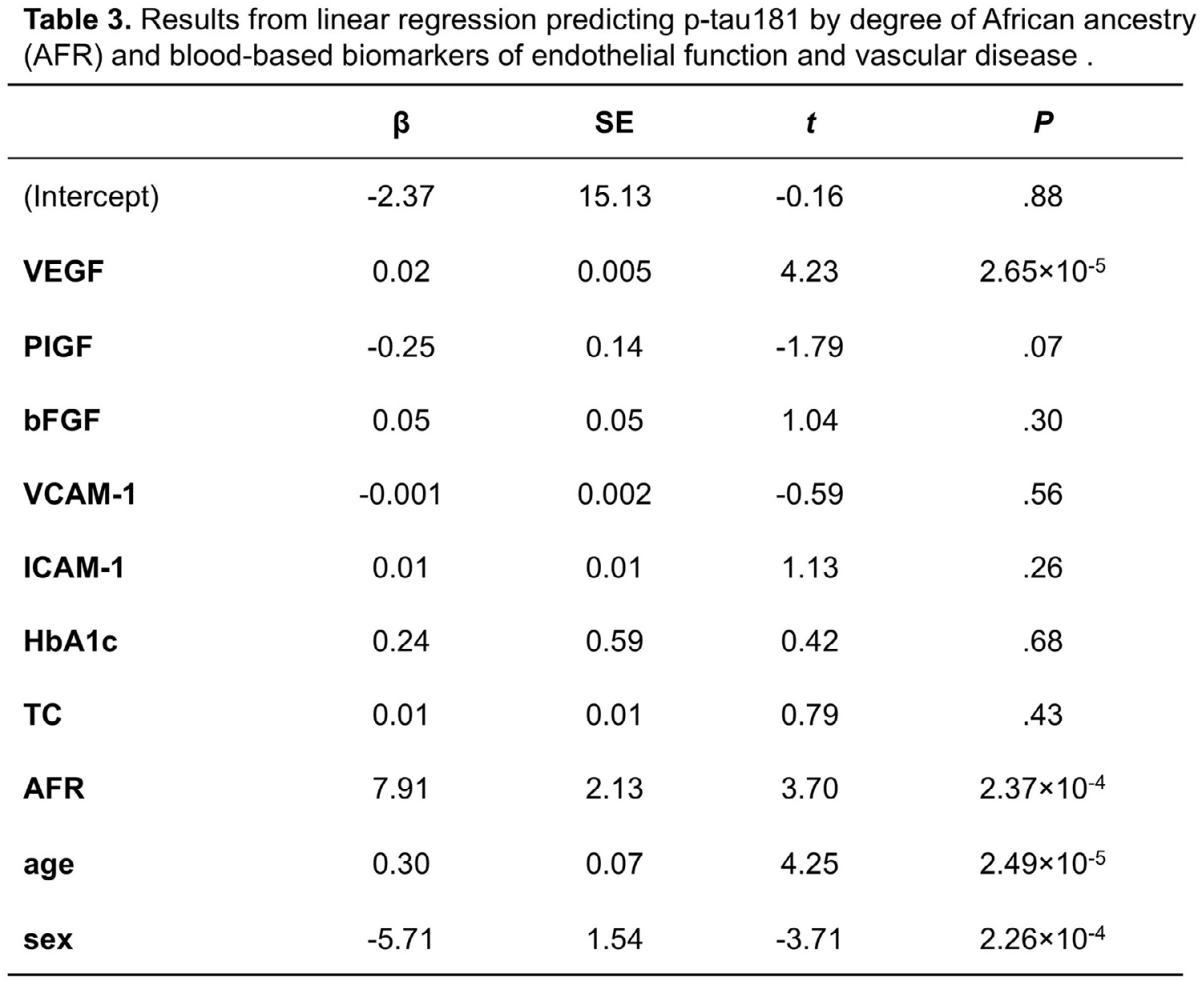# Associations of blood‐based biomarkers of vascular function with biomarkers of Alzheimer's Disease in individuals of admixed ancestry

**DOI:** 10.1002/alz70856_106480

**Published:** 2026-01-08

**Authors:** Nicholas R. Ray, Jiji T. Kurup, Jenny Chavez, Kara L Hamilton‐Nelson, Anthony J Griswold, Brian W Kunkle, William S Bush, Giuseppe Tosto, Adesola Ogunniyi, Rufus O Akinyemi, Jonathan L Haines, Goldie S Byrd, Jeffery M Vance, Margaret Pericak‐Vance, Christiane Reitz

**Affiliations:** ^1^ Columbia University Irving Medical Center, New York, NY, USA; ^2^ John P. Hussman Institute for Human Genomics, University of Miami Miller School of Medicine, Miami, FL, USA; ^3^ Hussman Institute for Human Genomics, University of Miami, Miller School of Medicine, Miami, FL, USA; ^4^ Department of Population & Quantitative Health Sciences, Case Western Reserve University School of Medicine, Cleveland, OH, USA; ^5^ Institute for Advanced Medical Research and Training, College of Medicine, University of Ibadan, Ibadan, Nigeria; ^6^ College of Medicine, University of Ibadan, Ibadan, Oyo, Nigeria; ^7^ Department of Population & Quantitative Health Sciences, School of Medicine, Case Western Reserve University, Cleveland, OH, USA; ^8^ Wake Forest University School of Medicine, Winston‐Salem, NC, USA; ^9^ University of Miami Miller School of Medicine, Miami, FL, USA

## Abstract

**Background:**

Poor cardiovascular health is a risk factor for cognitive decline and dementia, including Alzheimer's disease (AD). Development of ancestry‐informed blood‐based biomarkers for the small vessel diseases of the brain that contribute to vascular cognitive impairment across multiple populations is critical to identify individuals at risk. To begin addressing this, we examined the associations of blood‐based biomarkers of endothelial function and vascular disease with AD biomarkers and genetic ancestry in 1,534 admixed Hispanic and African American individuals from the PRADI and READD‐ADSP.

**Methods:**

Biomarkers of endothelial function and vascular disease included VEGF, PlGF, bFGF, VCAM‐1, ICAM‐1, HbA1C, and plasma lipid levels. AD biomarkers included Aβ40, Aβ42, and *p*‐tau181. Adjusting for age and sex as covariates, linear regressions were modeled separately predicting the three AD biomarkers by all 7 cardiovascular biomarkers, as well as degree of African ancestry. Additional regression models were run predicting each AD biomarker by each cardiovascular biomarker individually while controlling for age and sex and examining the interaction between the endothelial/vascular biomarker and African ancestry.

**Results:**

Aβ40 is associated with VEGF, PlGF, VCAM‐1, HbA1c, and African ancestry (Table 1); Aβ42 is associated with VEGF, PlGF, HbA1c, and African ancestry (Table 2); and *p*‐tau181 is associated with VEGF and African ancestry (Table 3). In addition, an interaction between cardiovascular biomarker and degree of African ancestry was observed for bFGF (β = 0.83, *P* = .03) while predicting Aβ40, ICAM‐1 (β = ‐0.006, *P* = .04) while predicting Aβ42, and VEGF (β = 0.03, *P* = .02) and total cholesterol (β = ‐0.11, *P* = .002) while predicting *p*‐tau181.

**Conclusions:**

Levels of VEGF, PlGF, VCAM‐1, and HbA1c predict levels of AD biomarkers in admixed individuals, and these associations are influenced by degree of African ancestry. While these findings need additional validation, they support the notion that endothelial disease contributes to cognitive impairment and that the assessed biomarkers may be valuable biomarkers for clinical settings. These results underscore the importance of biomarker research and validation in individuals from multiple populations.